# Development of genic KASP SNP markers from RNA-Seq data for map-based cloning and marker-assisted selection in maize

**DOI:** 10.1186/s12870-021-02932-8

**Published:** 2021-03-26

**Authors:** Zhengjie Chen, Dengguo Tang, Jixing Ni, Peng Li, Le Wang, Jinhong Zhou, Chenyang Li, Hai Lan, Lujiang Li, Jian Liu

**Affiliations:** 1 Maize Research Institute, Sichuan Agricultural University, 211 Huiming Road, Wenjiang District, Chengdu City, 611000 Sichuan China; 2 Industrial Crop Research Institute, Sichuan Academy of Agricultural Science, No.159 Huajin Avanue, Qingbaijiang District, Chengdu City, 610300 Sichuan China

**Keywords:** Maize, KASP, SNPs, High polymorphism, Molecular marker, RNA-Seq

## Abstract

**Background:**

Maize is one of the most important field crops in the world. Most of the key agronomic traits, including yield traits and plant architecture traits, are quantitative. Fine mapping of genes/ quantitative trait loci (QTL) influencing a key trait is essential for marker-assisted selection (MAS) in maize breeding. However, the SNP markers with high density and high polymorphism are lacking, especially kompetitive allele specific PCR (KASP) SNP markers that can be used for automatic genotyping. To date, a large volume of sequencing data has been produced by the next generation sequencing technology, which provides a good pool of SNP loci for development of SNP markers. In this study, we carried out a multi-step screening method to identify kompetitive allele specific PCR (KASP) SNP markers based on the RNA-Seq data sets of 368 maize inbred lines.

**Results:**

A total of 2,948,985 SNPs were identified in the high-throughput RNA-Seq data sets with the average density of 1.4 SNP/kb. Of these, 71,311 KASP SNP markers (the average density of 34 KASP SNP/Mb) were developed based on the strict criteria: unique genomic region, bi-allelic, polymorphism information content (PIC) value ≥0.4, and conserved primer sequences, and were mapped on 16,161 genes. These 16,161 genes were annotated to 52 gene ontology (GO) terms, including most of primary and secondary metabolic pathways. Subsequently, the 50 KASP SNP markers with the PIC values ranging from 0.14 to 0.5 in 368 RNA-Seq data sets and with polymorphism between the maize inbred lines 1212 and B73 in in silico analysis were selected to experimentally validate the accuracy and polymorphism of SNPs, resulted in 46 SNPs (92.00%) showed polymorphism between the maize inbred lines 1212 and B73. Moreover, these 46 polymorphic SNPs were utilized to genotype the other 20 maize inbred lines, with all 46 SNPs showing polymorphism in the 20 maize inbred lines, and the PIC value of each SNP was 0.11 to 0.50 with an average of 0.35. The results suggested that the KASP SNP markers developed in this study were accurate and polymorphic.

**Conclusions:**

These high-density polymorphic KASP SNP markers will be a valuable resource for map-based cloning of QTL/genes and marker-assisted selection in maize. Furthermore, the method used to develop SNP markers in maize can also be applied in other species.

**Supplementary Information:**

The online version contains supplementary material available at 10.1186/s12870-021-02932-8.

## Background

Maize (*Zea mays ssp. mays* L.) is one of the three most important cereal crops in the world, largely used as animal feed and source of industrial raw material. Maize has also been used as a classic model system in genetic research for over a century. In the face of rapidly growing populations and deteriorating environmental conditions, there is a pressing need to rapidly increase maize productivity through breeding. To date, the genetic cloning of key agronomic traits and marker-assisted selection (MAS) have been explored to enhance the efficiency of breeding programs. Molecular marker technology is a revolutionary technological advance in biological research and has become a critical tool for MAS. Over the past three decades, various types of molecular markers including AFLP, RFLP, RAPD, SSR, and InDel markers have been developed and successfully used in a wide range of applications in maize, [1–5]. Currently, geneticists and molecular breeders urgently need user-friendly, cost-effective and functional molecular markers that have high-density and high polymorphism.

SNPs are the most abundant source of genetic variation loci in plant genomes [6]. SNPs are now well recognized as the highest density molecular markers and are ideal for genetic studies. The use of markers with high coverage and high resolution can greatly improve the efficiency of genetic studies and plant breeding programs. The application of advanced SNP genotyping technology, such as next generation sequencing (NGS) and array-based genotyping, not only provides an efficient method for high- and medium-throughput SNP genotyping but also offers a lower cost per genotype [7–16]. Thus, the high-density SNP markers have been used widely in breeding and genomic studies, such as Genome Wide Association Study (GWAS), QTL mapping and germplasm resource evaluation. Fine mapping of QTL/genes and classical MAS techniques usually require the genotyping of a large number of samples (hundreds to thousands) at a few loci. However, the high-throughput genotyping platforms are not suitable for this type of study because of a high per sample cost. Fortunately, combining allele-specific oligo extension with fluorescence resonance energy transfer (FRET) for signal generation, KASP assays have emerged as an accurate, flexible and cost-effective SNP genotyping method [7]. Moreover, the KASP technology can be carried out in basic molecular laboratories. Additionally, the throughput of genotype calling is improved and the cost is significantly reduced due to the reduced reaction volume and the use of automated platforms.

In comparison to the whole genome sequencing (WGS) data, RNA-Seq data could provide a rich resource for the development of potential functional markers. For instance, a total of 5344 InDel markers were developed from the transcriptome data of sudangrass S722 and sorghum Tx623B [17], and 1276 polymorphic SSRs and 261,000 SNPs with functional meaning were identified based on the comprehensive transcriptome data of large yellow croaker [18]. Compared with traditional markers derived from random regions of the genome, genic markers have a greater potential to be associated with gene function. The application of genic markers in plant breeding could increase the selection efficiency by avoiding recombination between the markers and the gene coding for the trait of interest.

Furthermore, apart from the lack of high-density KASP SNP markers, another limitation to the application of KASP technology in map-based cloning of genes and MAS breeding programs is the success rate of PCR amplification, which can affect the performance of KASP SNP genotyping reactions. Therefore, there is an urgent need to close the knowledge gap between the large amounts of sequencing data and effective KASP SNP markers. In some cases, breeders and experimental researchers have struggled to handle the large amount of data generated through sequencing due to a lack of familiarity with the bioinformatic tools used to extract interesting information from the data. Additionally, the different types of molecular markers and methods used for genotyping have different properties. Therefore, the methods used to develop specific molecular markers must meet specific standards. After in silico analysis of the factors affecting the efficiency of SNP genotyping, the success rates and conversion rates will be improved, thus yielding accurate genotypes.

In recent years, KASP assays have been widely applied in maize studies on genetic diversity, genetic purity, quantitative locus mapping, QTL/genes fine mapping, marker-assisted selection, and marker-assisted breeding [19, 20]. For instance, 1057 KASP SNP markers were utilized to genotype 100 maize inbred lines, revealing a large number of genetic variations among these lines classified into four groups [21]. For quality control (QC) analysis of seed, 191 KASP SNP markers were used to evaluate the level of genetic purity among seeds of 16 maize inbred lines; the efficiency of 191 KASP SNP markers was close to that of 257,268 next-generation sequencing (GBS) markers with the correlation of 0.88, suggesting that smaller and high quality KASP SNP markers were sufficient for QC analysis [22]. Using the RNA-seq data of maize lines sensitive (LM11) and tolerant (CML25) to heat stress, 129,804, and 117,550 KASP SNPs were developed for LM11 and CML25, respectively; of which, 100 KASP SNPs were validated to genotype 90 F_2_ segregating population derived from LM11 × CML25 [23]. Moreover, the KASP SNP could serve as functional molecular markers. The KASP SNP (snpZM0015) located in the *crtRB1* gene affecting the content of provitamin A (PVA) effectively distinguished between favorable and unfavorable alleles in the 70 maize inbred lines [24]. Two SNPs located in the *GRMZM2G315401* and *GRMZM2G430362* were determined to be closely related to fertility through genotyping 50 sterile and 50 partially rescued plants by KASP assays [25]. Although many KASP SNP markers were developed in several maize inbred lines, the genome-wide high density and high polymorphism KASP SNP markers are limited.

In this study, to meet the requirements of genotyping fewer loci in a large number of samples, KASP SNP markers were developed based on the RNA-Seq data of developing kernels of 368 maize inbred lines. A set of 71,311 user-friendly KASP SNP markers with high-density and high polymorphism were identified, of which 50 KASP SNP markers were selected to experimentally validate the accuracy and polymorphism. The application of these high polymorphic markers will be helpful in enhancing the efficiency of map-based cloning and marker-assisted selection in maize.

## Results

### Characteristics of SNPs in the 368 RNA-Seq data sets

**Fig. 1:**
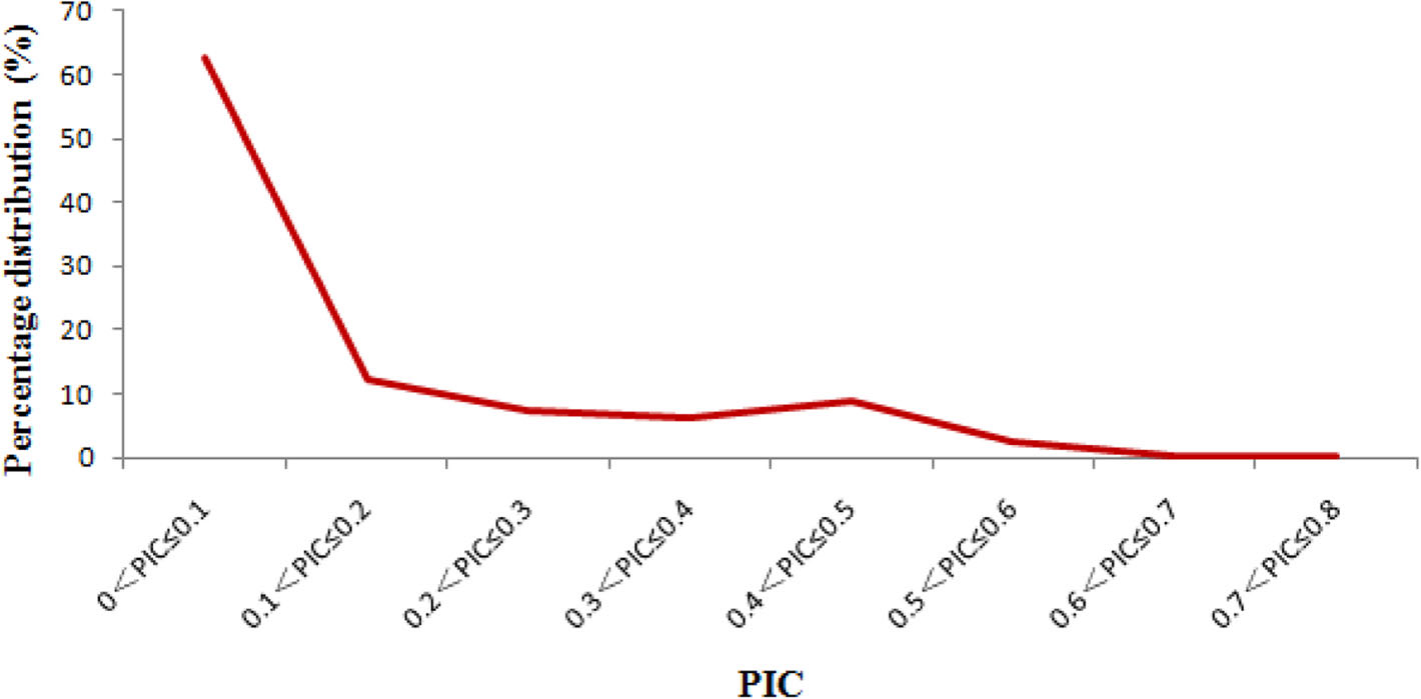
Distribution of the PIC values of SNPs

To mine SNPs in the RNA-Seq data sets of developing kernels of 368 maize inbred lines, resulted in a total of 2,948,985 SNP loci were identified. The number of SNP on individual chromosome ranged from 196,912 to 460,512, and the average density of SNPs in the maize genome was 1.4 SNPs/kb. Of these, 2,545,815 (86.33%) SNPs were mapped to the genic regions of 26,820 genes, with an average density of 94.92 SNPs/gene. Subsequently, the polymorphism information content (PIC) value of each SNP was calculated based on the genotypes of 368 maize inbred lines, and the PIC values ranged from 0.01 to 0.75, with an average of 0.13 (Fig. 1). The number of SNPs was drastically reduced with increasing PIC values, and the SNPs with the PIC values ≤0.1 accounted for 62.61%. Notably, there were 339,378 highly polymorphic SNPs (11.51%) with PIC values ≥0.4. In addition, the alleles for each SNP were counted. Most SNPs had two alleles (2,725,321), accounting for 92.42%; whereas 190,694 (6.47%) and 32,970 (1.12%) SNPs had three and four alleles, respectively. Because bi-allelic SNP markers are most widely used in genetic studies; the transition/transversion ratio of the bi-allelic SNPs was estimated, and the ratio of transition: transversion was 0.91:1. In addition, the most frequent type of transition was the T-to-C (28.79%), followed by A-to-G (28.36%), C-to-T (21.43%), and G-to-A (21.41%); the most abundant transversion was T-to-G (22.16%), followed by A-to-C (22.05%), G-to-C (14.71%), C-to-G (14.68%), G-to-T (8.31%), C-to-A (8.24%), T-to-A (5.01%), and A-to-T (4.84%).

### Development of highly polymorphic KASP SNP markers

**Fig. 2:**
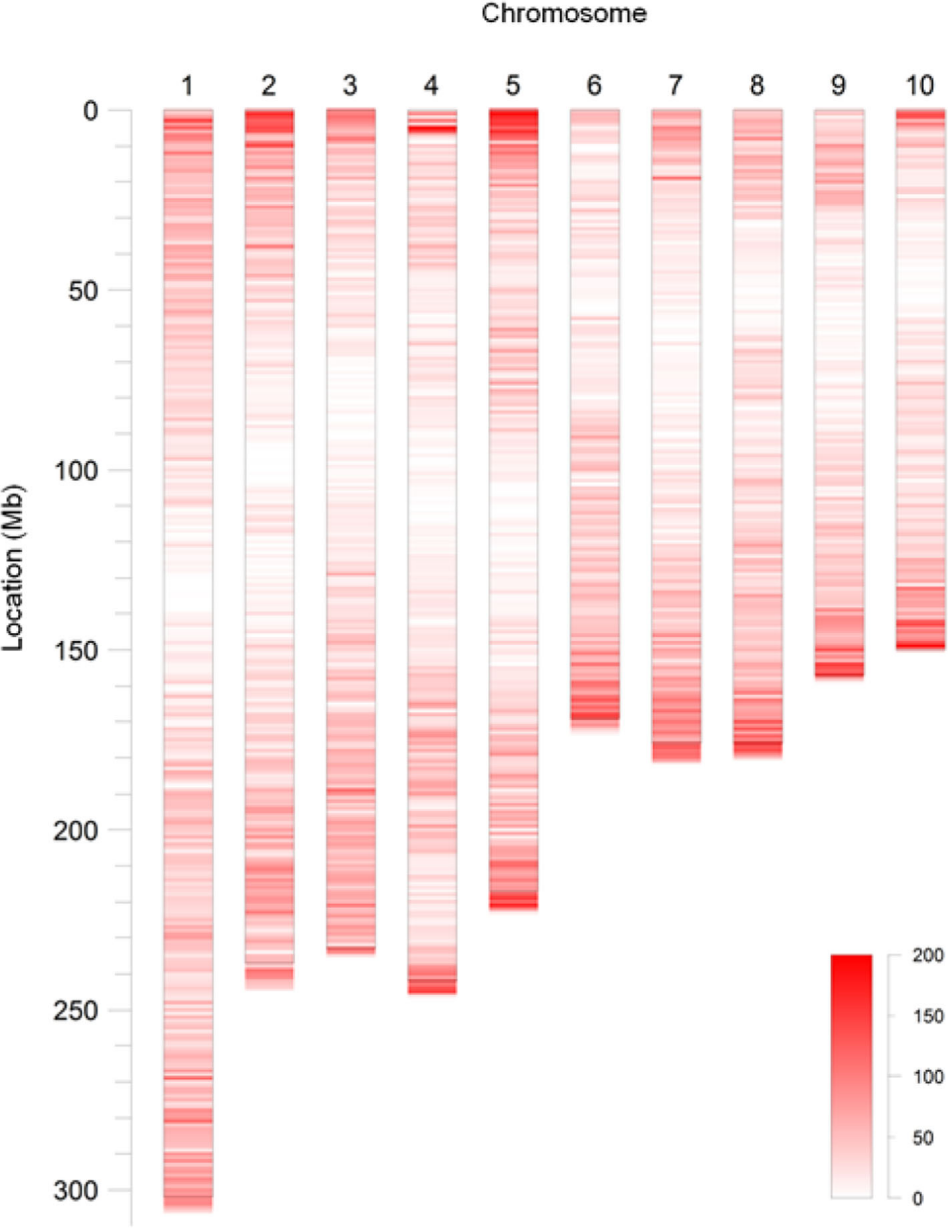
Heat map of density of 71,311 KASP SNP markers

In order to develop high quality and polymorphic KASP SNP markers, a fairly stringent set of criteria were applied, including SNP having a PIC value ≥0.4, a genotype missing rate ≤ 27.17% (≥100 genotypes), the presence of bi-allelic polymorphisms, unique primer sequences unfiltered by mapping the primer sequence to the reference genome with 1 mismatch, and highly conserved primer sequences among maize individuals (the PIC value of the SNP located within the KASP primer sequence was < 0.2). As a result, 71,311 KASP SNP markers were identified in 2,948,985 SNPs, which accounted for 2.42%. These KASP SNP markers were distributed on 10 chromosomes, ranging from 4678 to 11,756 on each chromosome. The highest density of KASP SNP occurred on chromosome 5 (39.26 SNP/Mb) and the lowest on chromosome 4 (25.77 SNP/Mb), and the average density was 33.40 SNP/Mb. In addition, the density of KASP SNP located at the end of the chromosomes was high, whereas near centromere it was low (Fig. 2). Of these 71,311 SNPs, 63,127 SNPs were located in the genic regions, accounting for 89.36%. The detailed information on SNPs, including the physical position of these SNPs on the maize genome (B73 RefGen v4.55 and v5), the PIC values, gene ID, GO annotations, SNP genotyping, allelic number, and number of samples with genotyping in the 368 maize inbred lines are provided in the Additional file 1.

Moreover, a core set of 8275 markers was selected by applying a more stringent set of criteria (Additional file 2). The first criterion was that the PIC values of SNPs located in the primer sequences were < 0.1; the second criterion was that the primer sequences needed to be mapped to unique genomic regions, allowing for ≤2 mismatches. Of these, the number of SNPs on each chromosome varied from 561 to 1374. These top level KASP SNP markers will be more user-friendly in future studies.

### GO annotation of genes associated withKASP SNP markers

**Fig. 3:**
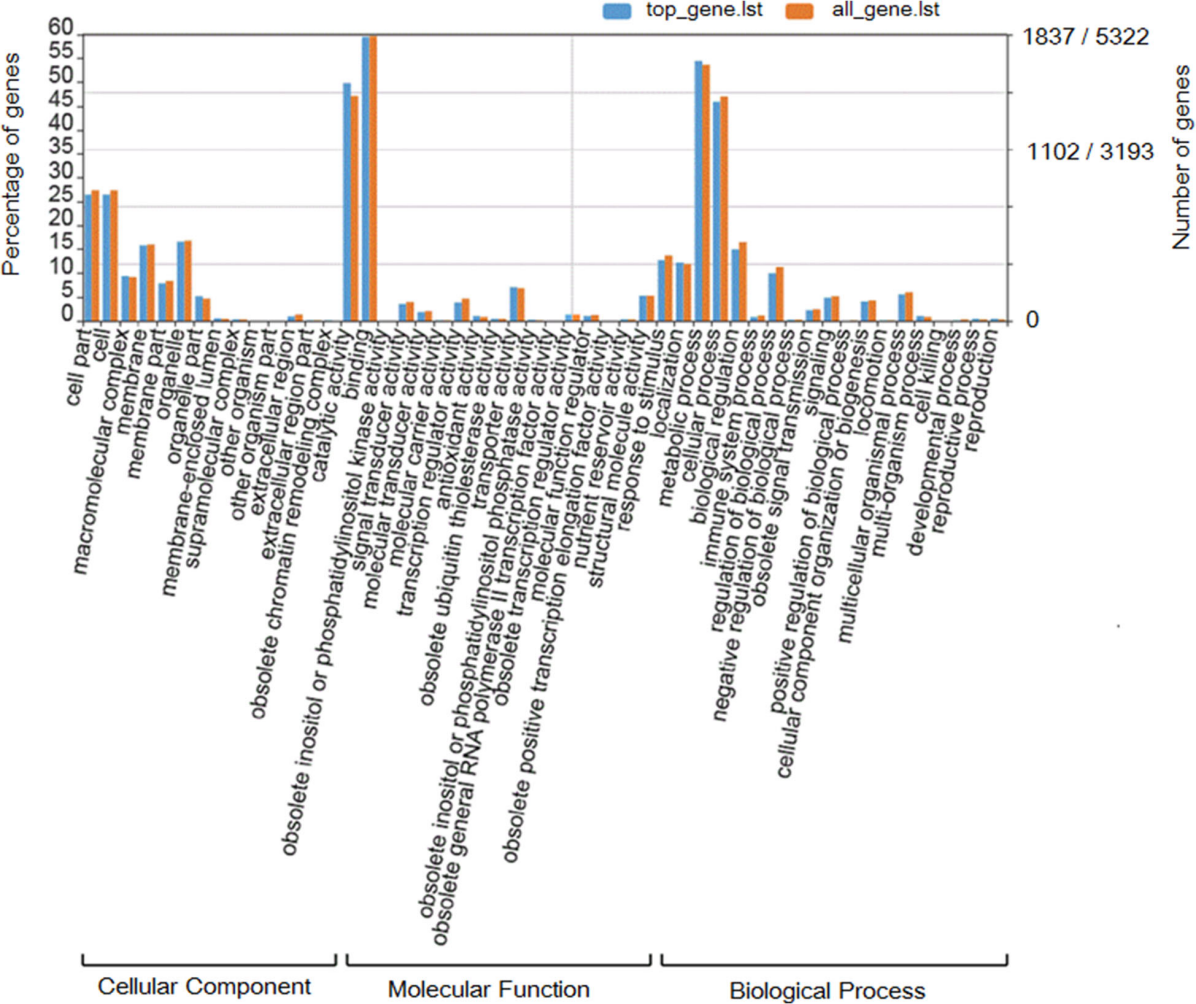
Summary of the functional analysis of GO terms. The numbers to the left and right of the slash ‘/’ represent the number of top gene and all gene, respectively

To explore the potential biological functions of these KASP SNP markers, GO enrichment analysis of the genes containing the KASP SNP markers was performed. Out of the 16,161 genes harboring at least one KASP SNP marker, 8870 genes were successfully classified into three categories including biological processes, molecular functions and cellular components using the AgriGO online tool for singular enrichment analysis (SEA) (Fig. 3; Additional file 3). Overall, 54 significant GO terms were identified at the second level, whereby 19 terms were associated with ‘biological processes’, 17 terms were related to ‘cellular components’ and 18 terms were involved in ‘molecular functions’, respectively. Among them, the predominant terms involved in the biological processes were ‘metabolic process’ (GO:0008152), ‘cellular process’ (GO:0009987) and ‘primary metabolic process’ (GO:0044238). The top three cellular components terms were ‘cell’ (GO:0005623), ‘cell part’ (GO:0044464), and ‘intracellular’ (GO:0005622). In the molecular functions category, ‘catalytic activity’ (GO:000382), ‘nucleotide binding’ (GO:0000166) and ‘hydrolase activity’ (GO:0016787) were the major GO terms. These results suggested that the genes involved in KASP SNP markers could influence most of metabolic pathways; thus, the KASP SNP markers could be widely utilized in studies of different traits and metabolic pathways.

### Validation of the accuracy and polymorphism of the KASP SNP markers

**Fig. 4:**
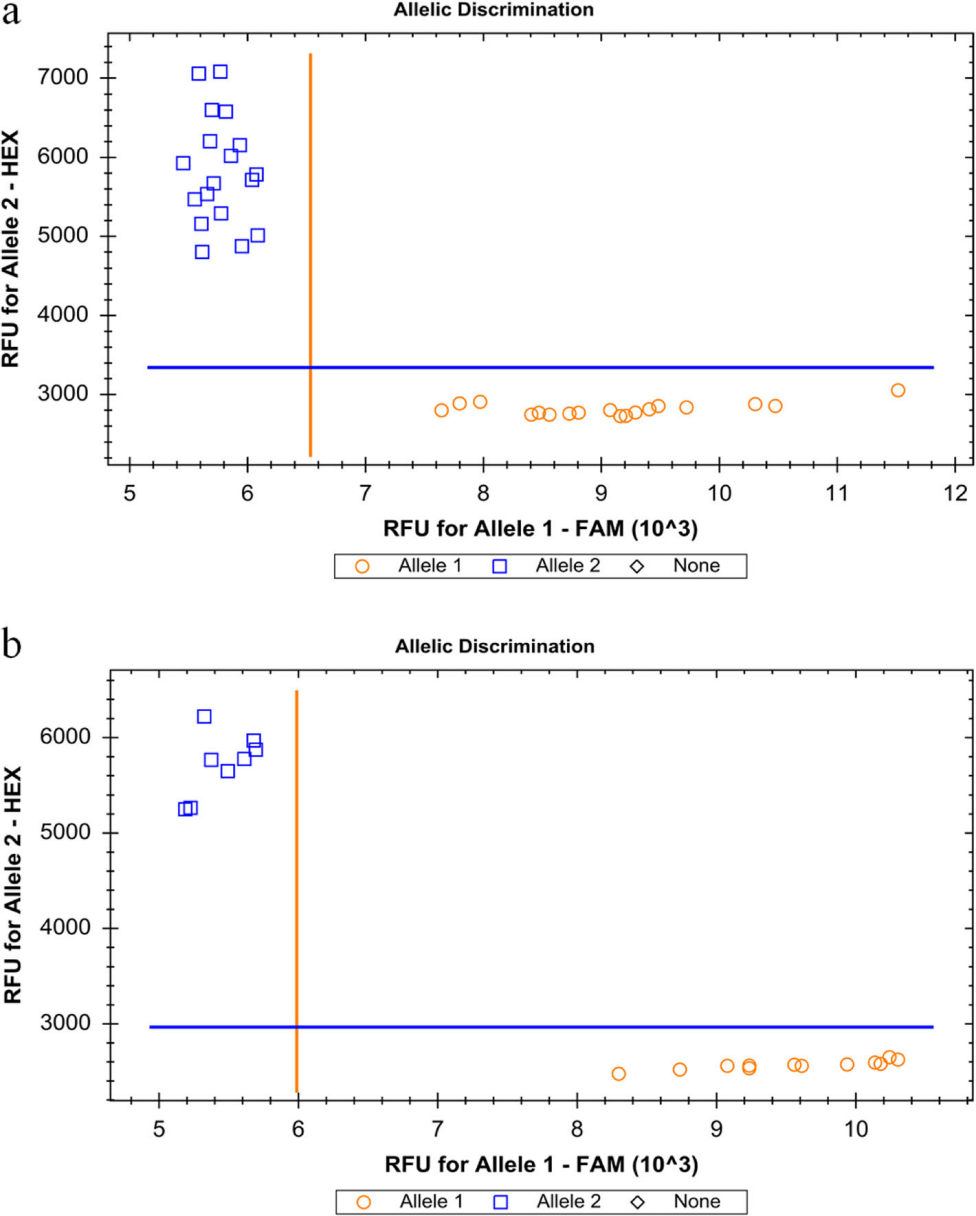
Genotype plot for the SNP markers using the KASP platform. **a** Genotype plot with three replicates for the SNP markers (from No.41 to No.47 in Additional file 4) in maize inbred lines 1212 and B73. **b** Genotype plot for the SNP marker Chr1–4,122,559 in the 20 maize inbred lines

To validate the accuracy and polymorphism of the KASP SNP markers, 50 KASP SNP markers with three to seven KASP SNP markers on each chromosome were randomly selected for PCR amplification detection. The PIC values of these 50 KASP SNP markers were 0.14–0.50 in the 368 RNA-Seq data sets; they all exhibited polymorphism between the maize inbred lines 1212 and B73 in in silico analysis (Additional file 4). Following validation, 46 (92.00%) out of 50 KASP SNP markers showed polymorphism between the maize inbred lines 1212 and B73 using PCR amplification, which was almost agreed with that of the in silico analysis. Subsequently, the polymorphic 46 KASP SNP markers were selected for genotyping of 20 maize inbred lines not part of the 368 lines used in the initial analyses. All 46 KASP SNP markers showed polymorphism among the 20 maize inbred lines. For each KASP SNP marker, the PIC value ranged from 0.10 to 0.50, with an average of 0.35 (Fig. 4; Additional file 4).

## Discussion

### The main advantages of the KASP genotyping technique

The KASP genotyping has several advantages compared to other SNP genotyping assays such as the TaqMan, high resolution melting (HRM) and matrix-assisted laser desorption/ionization time-of-flight mass spectrometry (MALDI-TOF-MS) [26, 27]. Firstly, the KASP genotyping technique is a cost-effective method of SNP genotyping. The cost per sample for KASP is less than that of most commonly used low throughput SNP genotyping methods mentioned above. A comparative study of SNP genotyping assays indicated that the cost per sample is 0.11 € for KASP compared to 0.29 € for TaqMan; further, the cost of commercial services for SNP genotyping is five times cheaper than that of TaqMan [28, 29]. Yuan et al. reported that the cost per data point of allele-specific KASP assay in soybean genotyping is about 1/50 of that of TaqMan [30]. Moreover, the use of miniaturized reactions of KASP SNP genotyping on an automated platform could further reduce the cost by up to 10 times. In addition, considering the number of markers and samples, KASP assays have the advantage over existing high-throughput SNP genotyping techniques, which have a higher cost per sample when genotyping a few loci in a large number of samples [31–33]. Secondly, KASP SNP genotyping is a simple gel-free fluorescent-based PCR assay. On the one hand, no specialized equipment is needed and researchers can carry out SNP genotyping using regular qPCR instruments. The SNPs could be converted to length polymorphisms by designing two primer pairs for different directional amplification of allele-specific SNP loci, such that DNA gel electrophoresis can be directly used to detect and separate the PCR products. Moreover, several simple ways could be chosen to perform KASP assays; for instance, the researchers could provide DNA samples and primers directly to LGC genomics offices or other commercial testing companies, or carry out KASP analyses in a qRT-PCR instrument through ordering KASP master mixes, or purchase the automated systems.

### Identification of highly polymorphic KASP SNP markers

The PIC value has been proven to be the most important indicator of the usefulness of molecular marker; the higher the PIC value is, the greater the probability of polymorphism between two arbitrary individuals is. Markers with the PIC values≥0.5 are usually considered highly informative [34, 35]. During the development of molecular markers, the accuracy of a PIC value for molecular marker is determined by the population size and its genetic structure. In this study, we employed the PIC value to evaluate the effectiveness of the SNP markers, and further ensured the accuracy of the PIC values using large-scale RNA-Seq datasets. The 368 maize inbred lines chosen to develop the KASP SNP markers in this study are fairly representative of global maize germplasms including temperate, subtropical and tropical materials; this dataset is suitable for avoiding a polymorphism bias. In contrast to SNP markers developed in the previous studies, the KASP SNP markers developed in this study have greater representation and high polymorphism levels. For example, the SNPs listed in the Panzea database were mainly developed from a relatively narrow dataset comprising of 14 maize varieties and 16 inbred lines of maize’s wild relative teosinte [36]. In another example, an in-depth analysis of the polymorphism level of the high-density maizeSNP50 array based on the same 368 maize inbred lines we used in the present study found that approximately half of the SNPs had PIC values lower than 0.3, and 877 SNPs were non-polymorphic markers [12, 37]. On the other hand, compared with other species, the average PIC value (0.13) of SNPs developed based on RNA-Seq data in this study was lower than the values in sesame (0.38) [38], radish (0.32) [39], grapevine (0.25) [40], and sweet orange (0.2) [41]. Considering the relatively low levels of polymorphism in the coding regions, the developed genic KASP SNP markers will be useful for map-based cloning and marker-assisted selection in maize.

### Optimizing the amplification efficiency of KASP SNP markers

Due to the sharply declining costs of NGS technology, millions of SNPs have been generated and published or deposited in public databases such as Panzea and MaizeGDB. To date, very little work has been done to comprehensively convert SNP markers to KASP markers in maize. Undoubtedly, not all SNPs meet the requirements of KASP SNP marker design [42]. The selection of SNP loci sets with high genotype call rate is thus the most important step in KASP SNP marker assays. Among the factors that influence the genotyping success rate, the primer sequence plays a key role in influencing the power of SNP genotyping in the KASP genotyping assays. In KASP assay primer design, the uniqueness and conservation of the primer sequences are the two most important parameters. The higher the degree of uniqueness and conservation a primer sequence has, the higher the SNP genotyping success rate is. This effect is particularly pronounced for species with a complex genome. For instance, because 85% of the maize genome is composed of repetitive sequences, it is difficult to avoid non-specific PCR amplification [43]. Therefore, we applied new methods and standards to increase the efficiency and accuracy of PCR amplification detection. PCR specific amplification was the prime factor that we considered in our pipeline. To improve the PCR specific amplification, the site-specificity of primer sequence for the KASP SNP marker was evaluated by aligning the primer sequence to the reference genome with ≤2 mismatches, and only the sequences mapped to unique genomic regions remained. The sequence conservation of KASP primer sequences was the second important factor we considered while developing the KASP SNP markers. Conservative primer sequences could increase the success rate of PCR amplification detection in different maize lines. In this study, two factors were utilized to screen for conserved primer sequences. One factor was the number of genotyping in 368 samples, and the threshold was set at ≥100. Another factor was the PIC value for the SNP located within a KASP primer sequence, such that the KASP primer sequences were removed when the PIC value was > 0.2. The success ratio of PCR amplification for 50 KASP SNP markers in the 20 inbred lines was 98.5%, suggesting that to optimize the parameters of SNP development, the amplification efficiency of KASP SNP markers was significantly improved.

### Development of user-friendly, high-density functional molecular markers

Compared to molecular markers randomly distributed on chromosomes, the genic KASP SNP markers developed in this study have several significant advantages in fine-mapping of QTL/genes and MAS [44]. As a genic SNP marker, the SNP variation derived from the coding region may change the structure of protein, which has a higher probability of being functionally linked to target traits. The 71,311 KASP SNP markers developed in this study were mapped to 16,161 genes with an average of 4.4 SNP markers per gene, which is approximately 116-fold higher than that of the gene-based KSAP SNP markers identified by Mammadov et al. in 2012 [42], and approximately 1.3-fold higher than that of maize 55 K SNP array with 19,425 SNPs in intronic regions developed by Xu et al. in 2017 [9]. Moreover, it is worth noting that these highly polymorphic genic KASP SNP markers were developed from a panel of 368 maize inbred lines. Of these, many inbred lines such as B73, S37 and 18–599, have been the parent lines of hybrids that are cultivated in large areas, and some of these lines have become key sources of germplasms in maize breeding programs. Overall, these KASP SNP markers not only could be used in map-based gene cloning, but also could be potentially a powerful tool for optimizing maize yield.

## Conclusions

Facing a high demand for genotyping several loci in a large number of samples in basic and applied research, our study provides a panel of 71,311 KASP SNP markers with an average of one KASP SNP marker per 29.94 kb that were identified with stringent selection criteria by combining RNA-Seq data of 368 maize inbred lines and bioinformatic methods. The accuracy ratio of the KASP SNP markers validated in the maize inbred lines 1212 and B73 reached 92.00%, and the PIC values among the 20 inbred lines were agreed with those of in silico analysis. Taken together, these KASP SNP markers will be beneficial for map-based gene cloning and MAS in maize research and the bioinformatics pipelines of marker development and screening parameters could be applied in other species.

## Methods

### Genome data and plant materials

The maize B73 reference genome (RefGen v4.55) and working gene set with annotated exon-intron structure were both downloaded from maizeGDB (http://www.maizegdb.org/assembly/) [42, 45]. The 368 RNA-Seq data of developing maize kernels were downloaded from the NCBI website (http://www.ncbi.nlm.nih.gov/sra/?term=SRP026161) and were utilized to mine SNPs (Additional file 5) [16].

The waxy maize inbred lines 1212 and B73 were selected for RNA-Seq analysis. The top of the immature kernels (20 DAP) from the 1212 and B73 were cut and snap frozen immediately in liquid nitrogen. Total RNA was isolated from each of the two samples using a Bioteke RNA extraction kit (Bioteke, Beijing, China) according to the manufacturer’s protocol, and RNA-Seq libraries were constructed according to the manufacturer’s recommendations. Both libraries were sequenced using a HiSeq2500 platform and each library produced more than 18 Gb of clean data. The RNA-Seq data of 1212 and B73 were used for in silico analysis.

Twenty maize inbred lines (SCML203, SCML202, SAM3001, PH6WC, CA1108, 2142, 891, 78,599–211, WA-1, YA8201, CA211, YA3237, SCML103, 21A, 9953, 08–641, LIAO6082, JH961, SN8–1-1, and 9614) selected from tropic and sub-tropic germplasms were used to evaluate the accuracy and polymorphism of KASP SNP markers.

### SNP mining and polymorphism evaluation of SNP loci

After filtering the low quality reads with < Q20 in the 368 RNA-Seq data sets using the NGSQC toolkit v2.3.3 [46], the clean reads were aligned to the maize B73 reference genome by BWA-ALN with the default parameters [47]. Then, the uniquely mapped reads were identified and used for further analysis. Later, genotype calling was evaluated using GATK (version 3.6–0) with the following filter conditions: QUAL ≥20 and coverage depth ≥ 5 [48]. The genotype set with missing data ≤5.43% (≤20 maize samples) were deleted. Finally, the allelic polymorphism of each SNP loci was assessed using the polymorphism information content (PIC) value, which was defined as PIC_*i*_ = 1- ${\sum}_{j=1}^n{p}_{ij}^2$, where p_*ij*_ was the frequency of the *j*th pattern for the *i*th marker [3]. In addition, the SNPs were also identified in RNA-Seq data of 1212 and B73 using the pipeline mentioned above.

### Identification of the unique and conserved primer sequences and design of primer sequences

The unique and conserved primer sequences were identified by the pipeline. First, 20 bp DNA sequences located upstream of the corresponding SNP locus were extracted using python scripts. Then, the 20 bp DNA sequences were aligned to the B73 reference genome using the Bowtie software with up to 1 mismatch [49]. Lastly, the sequences mapped to multiple genomic regions and containing SNPs with the PIC values > 0.2 were removed in the further analyses. Notably, the two allele-specific forward primers of the selected SNP markers were designed following the manual of KASP technology (LGC Genomics, Beverly, MA) [50], and the common reverse primer was designed using the Primer 3 software [51].

### GO analysis

Genes harboring KASP SNP markers were chosen for GO analysis. GO annotation and GO enrichment analyses were performed using the online agriGO GO Analysis Toolkit with the singular enrichment analysis (SEA) (http://bioinfo.cau.edu.cn/agriGO/index.php) [52], and the maize reference genome was used as the background reference. Hypergeometric tests with a Benjamini-Hochberg false discovery rate (FDR) were performed to adjust the *P*-value with the default parameters.

### Experimental validation of the accuracy and polymorphism of KASP SNP markers

A set of 50 bi-allelic SNPs was selected to validate the polymorphism level and accuracy of the KASP SNP markers. The genomic DNA was extracted from the 3-week-old seedlings of 22 maize inbred lines following the CTAB (cetyltrimethylammonium bromide) DNA extraction protocol. The PCR amplification for the SNP genotyping analysis was performed in a BioRad CFX-96 RT-PCR thermal cycler in a reaction mixture of 10 μL containing 1× KASP reaction mix, 0.17 μM KASP assay mix (two forward allele-specific primers and a common reverse primer) and 30–40 ng of genomic DNA. The reactions were performed with the following cycling conditions: an initial denaturation at 95 °C for 5 min, 35 cycles of denaturation at 95 °C for 30 s, annealing at 55 °C for 90 s, extension at 72 °C for 90 s, and a final extension at 72 °C for 10 min. Fluorescent SNP genotyping was analyzed using the CFX96 manager software. The PIC value for each SNP marker was calculated using the formula described above.

### Statement of methods

The authors declare that all methods complied with relevant institutional, national, and international guidelines and legislation.

## Supplementary Information

**Additional file 1.** The detailed information of 71,311 KASP SNP markers.

**Additional file 2.** The detailed information of the top 8,275KASP SNP markers.

**Additional file 3.** The GO annotations of the genes harboring KASP SNP markers.

**Additional file 4.** The results of validation of the accuracy and polymorphism of the 50 KASP SNP markers.

**Additional file 5.** The information of SNP mining in the 368 RNA-Seq data sets.

## Data Availability

All the data of this research are contained in the manuscript as well as in the supplementary files. All shell script program of this study is available from the corresponding author upon reasonable request.

## Abbreviations

KASP: Kompetitive allele-specific PCR
SNP: Single nucleotide polymorphism
InDel: Insertion and deletion
AFLP: Amplified fragment length polymorphism
RFLP: Restriction fragment length polymorphism
RAPD: Random amplified polymorphic DNA
SSR: Simple sequence repeat
MAS: Marker-assisted selection breeding
QTL: Quantitative trait loci
PIC: Polymorphic information content
GWAS: Genome-wide association study
GO: Gene ontology
